# Does the presence of cardiovascular disease risk factors or established disease influence the dietary intake of affected adults and their children residing in the same household? A secondary analysis of the Australian Health Survey (2011–2013)

**DOI:** 10.1186/s12872-017-0578-2

**Published:** 2017-06-05

**Authors:** Jolene Thomas, Lily Chan, Amanda Wray, Jacqueline Miller, Kaye Mehta, Alison Yaxley, Kacie Dickinson, Louisa Matwiejczyk, Kathryn Jackson, Michelle Miller

**Affiliations:** 1 Nutrition and Dietetics, Flinders University, Bedford Park, Adelaide, SA 5042 Australia; 2 Nutrition and Dietetics, Flinders University, GPO Box 2100, Adelaide, SA 5001 Australia

**Keywords:** Cardiovascular disease, Diet, Nutrients, Children, Sodium, Adults

## Abstract

**Background:**

Diet is an important contributor to risk of cardiovascular disease (CVD) and integral in management and delaying progression. Little is known however about whether increased CVD risk or established CVD has any influence on dietary intakes of Australian adults or children residing in the same household. This study aimed to determine whether the presence of CVD or CVD risk factors influences dietary intake of Australian adults and if the presence of an adult with increased CVD risk influences the dietary intake of a child living in the same household.

**Methods:**

Data were sourced from the 2011–2013 Australian Health Survey for: (1) adults ≥18 years with risk factors or established CVD and (2) children 2–17 years residing in the same household as adults with CVD risk factors or established CVD. Selected nutrient intakes (total fat, saturated fat plus trans fat, alpha-linolenic acid, total long chain omega 3 fatty acids, fibre and sodium) collected by repeated 24 h recalls were compared to national dietary recommendations and to the intakes of all other adults and children surveyed. Standard errors of the estimates were calculated using the replicate weights method, and an alpha value of <0.05 considered statistically significant.

**Results:**

Six thousand two hundred sixty five of 9435 adults surveyed were identified as having CVD risk factors or established disease and of these 1609 had a child in the same household that also contributed data in this survey. No differences were observed in adjusted mean dietary intakes between those without risk factors or established CVD and those with, except for total energy and sodium which were significantly lower in the adults with CVD risk factors and/or established disease. However sodium intakes across both groups were higher than recommended targets. There were no differences for selected nutrients between children residing with affected adults and other children surveyed.

**Conclusions:**

While intakes of Australian adults with CVD risk factors or established disease were favourable for sodium, compared to unaffected adults, there is still scope for improvement as many Australian adults, despite CVD risk, are unable to achieve targets for selected nutrients. Effective dietary behaviour change strategies and resources are urgently needed.

## Background

Cardiovascular disease (CVD), a major cause of mortality and morbidity worldwide, was responsible for 44,000 deaths in Australia in 2012 [1]. In 2010, CVD accounted for 25.6% of the burden of disease in Australia, second only to cancer [2]. While the cause of CVD can be multifactorial, a significant proportion of risk can be attributed to modifiable behavioural risk factors, the most important ones being smoking, poor nutrition, physical inactivity and excessive alcohol intake [1].

In an attempt to halt the rise in the prevalence of CVD and to reduce the risk of secondary complications of CVD, governments and public health agencies worldwide have developed evidence-based dietary recommendations and public health campaigns to target behavioural risk factors for CVD. In Australia, the Nutrient Reference Values for Australia and New Zealand (NRVs) provide recommended intakes of more the 35 macro-and macronutrients to meet adequate intakes for healthy individuals [3]. In addition, the NRVs also include a set of suggested dietary targets (SDTs) and acceptable macronutrient distribution ranges (AMDR) which provide nutrient recommendations for lowering chronic disease risk, including CVD. The Australian Dietary Guidelines (ADGs) also provide recommended intakes for optimal health and lowering chronic disease risk, in the form of meeting core groups rather than nutrients [4]. Furthermore the National Heart Foundation of Australia (NHF) has developed and/or endorsed evidence-based dietary recommendations for particular nutrients (and food groups) of relevance to CVD development and management, including total fats, saturated fats and trans fats combined, omega-3 fats, sodium, fibre and antioxidants [5–8].

There is a growing body of evidence indicating that dietary intake in childhood can influence CVD risk in adulthood. A review conducted in 2014 evaluated the evidence for the links between childhood and adolescent dietary patterns, dietary intakes and CVD risk in adulthood. The authors concluded that there were a limited number of longitudinal studies, nevertheless the literature supports the view that healthy childhood dietary patterns, particularly those rich in vegetables, fruits and fibre are associated with lower CVD risk in adulthood [9].

Information regarding diet and CVD is readily available to the general public via health professionals and a plethora of media, including, the internet and publications developed by agencies such as the NHF. Presumably adults with increased CVD risk or established disease will have had a degree of exposure to this information at some time point in the management of their condition. What is not well understood is whether dietary information and public health messages are translating into healthier dietary practices amongst individuals with CVD risk factors or diagnosed CVD.

It is well documented that parents/adult care providers and the family environment have a strong influence on the dietary intake and eating behaviours of children not only through the modelling of dietary practices and food habits, but also because they have direct control over foods available in the home [10–13]. It is also well documented that parental CVD risk has an influence on the CVD risk in later life of children [14]. What is not well understood is whether adults at risk of CVD or with established disease influence the dietary intakes of children residing in the same household.

The aim of this secondary analysis was to determine if the presence of CVD or CVD risk factors influences dietary intake of Australian adults and whether having an adult in the household with CVD or CVD risk factors influences the dietary intake of a child living in the same household utilising dietary intake data collected as part of the recent Australian Health Survey (AHS) [15].

## Methods

This is a secondary analysis of the AHS 2011–2013 [15]. The AHS 2011–2013 is the largest and most comprehensive health survey conducted in Australia to date. The data used in this analysis were collected during the National Nutrition and Physical Activity Survey 2011–2012 (NNPAS 2011–12), one of four components of the AHS 2011–2013. The NNPAS was conducted using a stratified multistage area sample of private dwellings, and within selected dwellings, a random sub-sample of residents was selected (one adult, and where applicable one child aged 2–17 years) [16]. Data collected provided information relating to dietary intakes (via two 24-h dietary recalls at least 8 days apart, using computer assisted interview instruments) and physical activity (via physical activity questionnaire and pedometer readings). One adult and one child aged 2 to 17 years, where applicable, from selected households were surveyed. Details of the survey can be found in the AHS Users’ Guide [16]. Permission was obtained from the Australian Bureau of Statistics (ABS) to access the basic confidentialised unit record file (CURF), released on November 13, 2014 to enable data analysis.

### Study population

#### Two-part analysis

Part 1: Comparison of dietary intake and proportion of adults (aged 18 years and over) meeting dietary recommendations between those with at least one CVD risk factor or established CVD and those without risk factors and established disease. Subjects were defined as having CVD risk factors if they were ever informed by a medical practitioner that they had: diabetes or high sugar level in blood/urine or high cholesterol or hypertensive disease, or were obese (body mass index ≥30 kg/m^2^) or had a waist circumference ≥ 80 cm for females and ≥94 cm for males. Subjects were defined as having established CVD if they reported ever having angina, ischaemic heart disease, cerebrovascular disease, heart failure or other heart disease.

Part 2: Comparison of dietary intake and proportion of children (aged 2–17 years) meeting recommendations between those children living in the same household with the adults identified in Part 1 (i.e. those with at least one CVD risk factors or established CVD) and the rest of the children in the survey. Survey subjects from the same household shared the same Identification Number except for the last digit thereby enabling the matching process.

#### Estimation of usual intakes

Data from the first 24-h dietary recall conducted at the face-to-face interview and the second telephone interview were used to estimate usual intakes. To ensure consistent and complete data collection, 24-h dietary recall was conducted by trained interviewers using a computer-assisted interview system, the Automated Multiple-Pass Method [17]. The Food Standards Australia New Zealand AUSNUT 2011–2013 food nutrients database developed for this survey was used to estimate nutrients from all food and beverages, excluding supplements, consumed during the previous 24-h period. Dietary intake of the following nutrients were examined in this analysis as they were specifically related to CVD: total fat, saturated fat plus trans fat, alpha-linolenic acid (ALA), total long chain omega 3 fatty acids (LCN3), fibre and sodium. LCN3 intake in the form of supplements was also investigated to determine if there was a difference in supplemental LCN3 intake in adults with higher CVD risk. The consumption of alcohol was also examined. Intake recommendations were based on the National Health and Medical Research Council (NHMRC) Nutrient Reference Values for Australia and New Zealand document [3] as well as the National Guideline for Alcohol Consumption [18].

#### Statistical analysis

STATA version 10.1 (StataCorp LP, College station, TX, USA) was used to compare the demographics between the groups. Usual intakes distribution and the proportion of the population meeting requirements according to the National Cancer Institute (NCI) method [19] was estimated using SAS for Windows 9.4. For the analysis with additional adjustment for covariates, SAS Macros Version 2.1, Mixtran and Distrib, were downloaded from the NCI website [20]. Covariates included in the modelling were age group, gender, Socio-Economic Indexes for Areas (SEIFA) quintiles (1st quintile was the lowest 20% and 5th quintile was the highest 20%), smoking status (Current smoker, Ex-smoker, Never smoked), dietary intakes on weekday (Monday to Friday) or weekend (Saturday and Sunday) day, and the sequence of dietary recalls (first or second 24-h recall). Recall sequence accounts for the differences in reported intakes which tend to occur depending on whether it was the first or second report from the individual. Since a large proportion of subjects did not report any alcohol consumption, a two-part correlated model was adopted for the estimation of alcohol intake which involved both the estimation of the probability of consuming and the amount of alcohol consumed [21]. For other reported nutrients that were consumed almost every day by nearly everyone, a one-part model was used where the probability of consuming was assumed to be one and therefore only the amount consumed was modelled. Means and proportions were estimated with the sampling weights (provided with the data set) applied in order to compensate for unequal probability of selection of subjects, non-response and non-coverage. For all analysis, the replicate weights methodology was used to calculate the standard errors of the estimates [22]. Briefly, this involved running the analysis repeatedly using the 60 replicate weights (obtained using delete-a-group jack-knife method) provided in the data set. The distribution of this set of replicate estimates, in conjunction with the full sample estimate was then used to approximate the standard errors of the full sample. Chi-squared test was used to determine if there were demographic differences between the groups. For intake comparisons, if the standardised difference between two groups was less than −1.96 or greater than 1.96, that difference was reported as significant at α = 0.05 level [22].

## Results

**Fig. 1:**
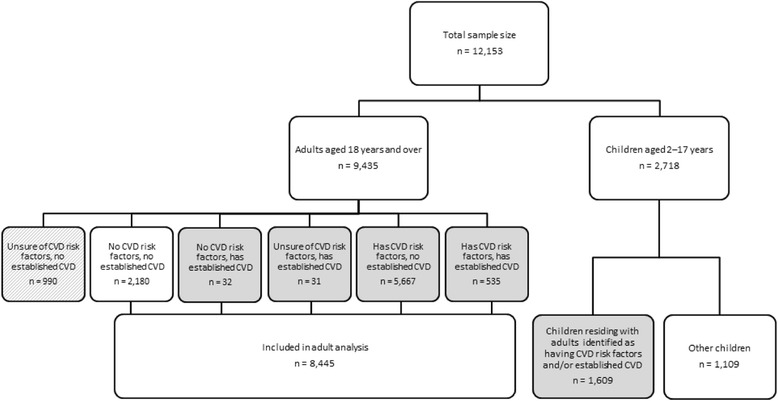
Selection of adult subjects with cardiovascular disease (CVD) risk factors* or established CVD** (adult analysis) and children residing in the same household (children analysis) from the total sample of the Australian Health Survey (2011–2013). *CVD risk factors: Diabetes, high sugar level in blood/urine, high cholesterol, hypertensive disease, obese (BMI ≥30 kg/m2), waist circumference ≥ 80 cm in female or ≥94 cm in male. **Established CVD: Angina, Ischaemic heart disease, cerebrovascular disease, heart failure or other heart disease

Figure 1 provides an overview of the selection process followed to produce the adult and children groups included in this analysis.

### Adult dietary intake

Adult participant characteristics are described in Table 1. Of the 8445 adults included in the analysis, 2180 were deemed to have no CVD risk factors or established CVD and 6265 had at least one CVD risk factor and/or established disease. The gender distribution was significantly different between the groups with more females in the group with CVD risk factors and/or established disease (50.9%) compared to the non-risk group (45.3%) (*p* < 0.001) and as expected, there was a significantly higher proportion (*p* < 0.001) of adults in the older age groups in the group with CVD risk factors and/or established CVD (50.3% aged 51 year or above) compared to the non-risk group (15.7% aged 51 year or above). There was a significantly higher proportion of adults who had never smoked in those with no CVD risk factors or established CVD and a higher proportion of ex-smokers in adults with CVD risk factors and/or disease. There was a greater proportion of adults with CVD risk factors and/or disease from lower socioeconomic groups compared to those with no CVD risk factors or established disease.

**Table 1:** Characteristics of adult participants according to their health status, i.e. those with no cardiovascular disease (CVD) risk factors or established CVD vs. those with at least one CVD risk factor or established CVD) in the National Nutrition and Physical Activity Survey, 2011–12

Characteristics	No risk factor or CVD (*n* = 2180) n (weighted %, 95% CI)	Has at least one risk factor and/or CVD (*n* = 6265) n (weighted %, 95% CI)	*p*-value
Gender			
Male	1135 (54.7%, 52.6–56.8%)	2851 (49.1%, 48.1–50.1%)	*p* < .001
Female	1045 (45.3%, 43.2–47.4%)	3414 (50.9%, 49.9–51.9%)	
Age (Years)			
18–30	804 (47.7%, 45.8–49.5%)	682 (14.3%, 13.3–15.4%)	*p* < .001
31–50	940 (36.7%, 34.9–38.5%)	2159 (35.4%, 34.5–36.4%)	
51–70	367 (13.4%, 11.9–15.0%)	2297 (35.4%, 34.3–36.5%)	
> 70	69 (2.3, 1.7–3.1%)	1127 (14.9, 14.4–15.3%)	
Smoking now			
Current smoker	441 (18.6%, 16.6%–20.7%)	1143 (17.4%, 16.2%–18.6%)	*p* < .001
Ex-smoker	521 (22.3%, 20.3%–24.3%)	2269 (35.0%, 33.3%–36.7%)	
Never smoked	1218 (59.1%, 56.5%–61.6%)	2853 (47.7%, 46.1%–49.3%)	
SEIFA (Index of Relative Socio-Economic Disadvantage 2011			
Lowest 20%	344 (15.2%, 12.5%–18.2%)	1260 (19.5%, 17.4%–21.8%)	*p* < .001
Second quintile	373 (17.5%, 15.1%–20.2%)	1353 (20.8%, 18.9%–22.9%)	
Third quintile	435 (20.2%,17.1%–23.6%)	1260 (21.4%, 19.3%–23.6%)	
Fourth quintile	399 (19.9%, 16.5%–23.9%)	1097 (18.3%, 15.9%–20.9%)	
Highest 20%	629 (27.3%, 24.0%–30.9%)	1295 (20.0%, 18.0%–22.2%)	
Consumed total LCN3^a^supplements supplements ^a^ (either	15.9% (13.9%–18.1%)	17.8% (16.6%–19.1%)	*p* = 0.123

*Reported to have taken total long chain omega 3 fatty acids (LCN3) supplements on either day 1 or day 2 of survey or on both days

The usual dietary intake of selected nutrients, adjusted for covariates, of the 2180 adult subjects with no CVD risk factors or established CVD and the 6265 adult subjects with at least one CVD risk factor or established CVD are presented in Table 2. Those with at least one CVD risk factor or established CVD were found to have a significantly lower intake of total energy (kJ), total fat (g), saturated/trans fat combined (g), α-linolenic acid (ALA, g), fibre (g) and sodium (mg) when compared to those with no CVD risk factors or established CVD. There was no difference in total fat, saturated/trans fat combined, long-chain omega three fats (LCN3), α-linolenic acid intake or fibre when adjusted for energy intake. Mean alcohol intake was found to be similar across the two adult groups. Sodium intake was significantly lower in the adults with CVD risk factors and/or established CVD after adjusting for energy intake (2418 mg vs 2576 mg), however intakes were still higher than the recommended target of <5 g salt (<1938 mg sodium) [23] or <4 g salt (<1550 mg sodium) [24] per day for primary and secondary prevention of CVD respectively.

**Table 2:** Comparison of usual dietary intake (from food only) between adults with and without CVD/risk factors^a^

Nutrient / Alcohol	Mean (95% Confidence Intervals)		Test statistics^b^
	No risk factor or CVD (*n* = 2180)	Has at least one risk factor and/or CVD (*n* = 6265)	
Total energy (kJ)	9351 (9167–9536)	8521 (8379–8664)	6.98*
Total fat (g)	80.3 (78.0–82.6)	72.6 (71.1–74.1)	5.46*
Total fat as percentage of total energy intake (%)	31.1 (30.6–31.6)	30.8 (30.5–31.1)	1.13
Saturated +Trans fat (g)	31.5 (30.4–32.6)	28.7 (28.0–29.4)	4.26*
Saturated +Trans fat as percentage of total energy intake (%)	12.2% (11.9%–12.4%)	12.1% (11.9%–12.3%)	0.25
Alpha-linolenic acid (g)	1.49 (1.44–1.55)	1.39 (1.35–1.43)	2.83*
Alpha-linolenic acid as percentage of total energy intake (%)	0.59 (0.57–0.61)	0.60 (0.59–0.61)	−0.95
Total long chain omega 3 fatty acids (mg)	257.8 (238.9–276.7)	247.4 (232.3–262.6)	0.83
Fibre (g)	24.2 (23.5–24.9)	22.5 (22.0–22.9)	4.22*
Fibre (g/MJ of energy)	2.7 (2.7–2.8)	2.8 (2.7–2.8)	−1.41
Sodium (mg)	2576 (2496–2656)	2418 (2365–2471)	3.22*
Sodium (mg/MJ of energy)	284 (279–290)	292 (288–297)	−2.22*
Standard drink (10 g of alcohol per drink)	1.3 (0.7–1.9)	1.7 (1.1–2.2)	−0.79

^a^ Usual intake estimated from two 24-h dietary recalls, adjusted for sequence (from personal interview or telephone interview), day of week (weekdays, Mon to Fri; or weekend days, Sat and Sun), age groups (18; 19–30; 31–50; 51–70, or >70 years), gender, SEIFA quintiles (where 1st quintile was the lowest 20% and 5th quintile was the highest 20%), smoking status (current smoker, ex-smoker, never smoked)

^b^ If the test statistic was <−1.96 or >1.96, the difference between the two groups was considered significant at the α = 0.05 level and marked with an asterisk

When the proportion (% ± 95% CI) of adults in both groups meeting the dietary recommendations for the nutrients of interest was investigated (Table 3), no significant differences were observed between the two adult groups except for sodium (no risk/disease 7.8% (5.7–9.8%) versus affected adults 11.9% (9.6–14.2%) *p* < 0.05) and fibre (no risk/disease 13.4% (11.3–15.5%) versus affected adults 9.9% (8.4–11.4%), *p* < 0.05) however the proportions meeting the recommendations for these nutrients were very low in both groups. Adults in both groups were more successful in meeting the recommendations for total fat intake and ALA intake with over 80% of adults meeting these targets, however the proportion of adults meeting the LCN3 (from food only) recommendations were very low at 4.8% (95%CI 2.6–7.1%) and 4.3% (95% CI 2.1–6.4%). Approximately three-quarters of adults met the recommendations for alcohol consumption.. The proportion of adults who had consumed LCN3 supplements on either day one or day two of the survey or on both days was also similar across both groups with 15.9% (95% CI 13.9–18.1%) of adults in with risk or disease and 17.8% (95% CI 16.6–19.1%) of affected adults consuming supplements (*p* = 0.123).

**Table 3:** Proportion^a^ of adults with and without CVD/risk who meet recommendations according to the NH&MRC^b^ guidelines

Nutrient / Alcohol	Recommended intake (per day on average)	Proportion (95% CI) meeting recommendations		Test statistics
		No risk factor or CVD (*n* = 2180)	Has at least one risk factor +/− CVD (*n* = 6265)	
Total fat (AMDR)	20–35% of total energy intake	82.1% (78.3%–86.0%)	83.9% (80.9%–87.0%)	−0.72
Sat fat + Trans fat (AMDR)	≤10% of total energy intake	18.9% (15.1%–22.8%)	19.6% (16.9%–22.3%)	-0.28
ALA (AMDR)	0.4–1% of total energy intake	86.6% (82.7%–90.5%)	87.6% (84.1%–91.1%)	−0.35
LCn-3PUFA (SDT)	Male 610 mg Female 430 mg	4.8% (2.6%–7.1%)	4.3% (2.1%–6.4%)	0.34
Fibre (SDT)	Male 38 g Female 28 g	13.4% (11.3%–15.5%)	9.9% (8.4%–11.4%)	2.60*
Sodium (SDT)	≤1600 mg/day	7.8% (5.7%–9.8%)	11.9% (9.6%–14.2%)	−2.59*
Standard drinks	≤2 standard drinks	76.1% (64.4%–87.8%)	70.6% (61.0%–80.1%)	0.72

^a^ Proportion estimated from two 24-h dietary recalls and from food intake only, adjusted for sequence (from personal interview or telephone interview), day of week (weekdays, Mon to Fri; or weekend days, Sat and Sun), age groups (18; 19–30; 31–50; 51–70, or >70 years), gender, SEIFA quintiles (where 1st quintile was the lowest 20% and 5th quintile was the highest 20%), smoking status (current smoker, ex-smoker, never smoked)

^b^ Recommendation according the NH&MRC, Optimising diets for lowering chronic disease risk (AMDR – Acceptable Macronutrient Distribution Range; SDT – Suggested Dietary Targets) and for alcohol, national guidelines for alcohol consumption where one standard drink equals to 10 g of pure alcohol

^c^ If the test statistic was <−1.96 or >1.96, the difference between the two groups was considered significant at the α = 0.05 level and marked with an asterisk

Further analysis exploring the intake of ALA and EPA + DHA in adults with CVD risk factors and/or established CVD showed only 13.3% (95% CI 10.5–15.2%) were meeting the increased recommendation of >2 g/day of ALA and 0.02% (95% CI 0–0.08%) were meeting the ≥1 g/day eicosapentaenoic acid + docosahexaenoic acid (EPA + DHA) target from food [24]. When combined with the proportion of adults with CVD risk factors and/or established CVD consuming LCN3 in the form of supplements [15.9% (95% CI 13.9–18.1%)] it would be safe to assume that the majority of adults in this group would not be meeting the recommendations for LCN3 intake.

### Children residing in the same household as an adult with CVD and/or CVD risk factors

Characteristics of the two groups of children, those living with an adult with CVD risk factors and/or established CVD and all other children are displayed in Table 4. There was a difference in the gender distribution between the groups with a higher proportion of boys in the group of children living in a household with a CVD adult (*p* = 0.045). There was no statistically significant difference in the age group distribution or mean age of the two groups (Table 4).

**Table 4:** Characteristics of children participants according to their living environment, i.e. those from household with adults known to have cardiovascular disease (CVD) or risk factor vs. those without, in the National Nutrition and Physical Activity Survey, 2011–12

Characteristics	Children living in CVD households (*n* = 1609)	Other children (*n* = 1109)	*p*-value
	n (weighted %, 95% CI)	n (weighted %, 95% CI)	
Gender			
Boy	833 (53.0%, 51.4%–54.7%)	540 (48.8%, 46.3%–51.3%)	=0.045
Girl	776 (47.0%, 45.3%–48.7%)	569 (51.2%, 48.8%–53.7%)	
Age (Years)			
2–3	261 (11.8%, 10.1%–13.6%)	203 (13.7%, 11.7%–15.9%)	=0.189
4–8	461 (29.4%, 26.4%–32.7%)	328 (32.4%, 28.5%–36.5%)	
9–13	495 (36.1%, 32.7%–39.7%)	292 (30.9%, 27.1%–35.0%)	
14–17	392 (22.7%, 20.3%–25.3%)	286 (23.1%, 20.5%–25.9%)	

The estimated mean usual intakes of key nutrients in these children were compared to other children surveyed that did not reside with an adult with CVD and/or CVD risk factors (*n* = 1109) and no significant differences were observed (Table 5).

**Table 5:** Comparison of usual dietary intake^a^ (from food only) in children between those from household with adults known to have cardiovascular disease (CVD) or risk factor vs. those without (other children)

Nutrient	Mean (95% Confidence Intervals)		Test Statistics^b^
	Children living in households with known adults with CVD or CVD risk factors (*n* = 1609)	Other children (*n* = 1109)	
Total fat as a % of total energy intake	31.1% (30.6–31.6%)	31.1% (30.6%–31.7%)	−0.02
Saturated fat + Trans fat as a % of total energy intake	13.7% (13.4%–14.0%)	13.7% (13.3%–14.1%)	0.20
Alpha-linolenic acid	1.11 (1.06–1.17)	1.13 (1.08–1.18)	0.40
Total long chain omega 3 fatty acids	115.8 (107.7–124.0)	125.7 (113.4–138.1)	1.31
Fibre	19.5 (18.9–20.1)	19.6 (19.0–20.3)	0.30
Sodium	2300 (2222–2378)	2259 (2187–2331)	−0.75

^a^ Usual intake estimated from two 24-h dietary recalls, adjusted for sequence (from personal interview or telephone interview), day of week (weekdays, Mon to Fri; or weekend days, Sat and Sun), age groups (18; 19–30; 31–50; 51–70, or >70 years) and gender

^b^ All test statistics were either > − 1.96 or <1.96, therefore none of the differences between the two groups were considered significant at the α = 0.05 level

Similar results were observed when the proportion of children meeting recommendations of selected nutrients was explored with no differences found between the two groups of children (Table 6). However the majority of children living in a household with an adult with CVD risk/established disease and all other children respectively [90.1% (95% CI 84.9–95.2%) and 90.1 (95% CI 85.1–95.0)] were meeting the AMDRs for total fat intake, ALA [80.8% (74.2–87.5%) and 83.1% (76.2–90.0%)] and total long-chain omega-3 fats [89.2% (81.0–97.5%) and 93.4% (85.6–100.0%)]. A low proportion of children met the recommendations for saturated and trans fats [3.2% (95% CI 0.9–5.6%) and 3.0% (95% CI 0.6–5.5%)] and sodium [17.5% (95% CI 12.5–22.5%) and 17.2% (95% CI 12.4–22.0%)].

**Table 6:** Proportion^a^ of children who met recommendations according to the NH&MRC^b^ guidelines

Nutrient	Recommended intake (per day on average)	Proportion (95% CI) meeting recommendations		Test Statistics
		Children living in households with known adults with CVD or CVD risk factors (*n* = 1609)	Other children (*n* = 1109)	
Total fat (AMDR)	20–35% of total energy intake	90.1% (84.9%–95.2%)	90.1% (85.1%–95.0%)	0.003
Sat fat + Trans fat A(AMDR)	≤10% of total energy intake	3.2% (0.9%–5.6%)	3.0% (0.6%–5.5%)	-0.11
ALA (AI)	1–3 years 0.5 g 4–8 years 0.8 g Boys 9–13 years 1 g 14–18 years 1.2 g Girls 9–13 years 0.8 g 14–18 years 0.8 g	80.8% (74.2%–87.5%)	83.1% (76.2%–90.0%)	0.47
Total LC N3 (AI)	1–3 years 40 mg 4–8 years 55 mg 9–13 years 70 mg Boys 14–18 years 125 mg Girls 14–18 years 85 mg	89.2% (81.0%–97.5%)	93.4% (85.6%–100.0%)	0.72
Fibre (AI)	1–3 years 14 g 4–8 years 18 g Boys 9–13 years 24 g 14–18 years 28 g Girls 9–13 years 20 g 14–18 years 22 g	40.8% (36.7%–44.9%)	43.6% (39.0%–48.1%)	0.88
Sodium (UL)	1–3 1000 mg 4–8 years 1400 mg 9–13 years 2000 mg 14–18 years 2300 mg	17.5% (12.5%–22.5%)	17.2% (12.4%–22.0%)	−0.08

^a^ Proportion estimated from two 24-h dietary recalls and from food intake only, adjusted for sequence (from personal interview or telephone interview), day of week (weekdays, Mon to Fri; or weekend days, Sat and Sun), age groups (18; 19–30; 31–50; 51–70, or >70 years), gender, SEIFA quintiles (where 1st quintile was the lowest 20% and 5th quintile was the highest 20%), smoking status (current smoker, ex-smoker, never smoked)

^b^ Recommendation according the NH&MRC, Optimising diets for lowering chronic disease risk (AMDR – Acceptable Macronutrient Distribution Range for total fat, saturated plus trans fat); adequate intake (AI) for total long chain omega 3 fatty acids (LCN3) and fibre; upper level of intake (UL) for sodium

^c^ All test statistics were either > − 1.96 or <1.96, therefore none of the differences between the two groups were considered significant at the α = 0.05 level

## Discussion

This secondary analysis of the AHS [15] found very little difference in the average dietary intakes of Australian adults with CVD risk factors and/or established CVD and those without CVD risk factors. The exception was sodium intake, which was significantly lower (*p* < 0.05) in adults with CVD risk factors and/or established CVD even after adjusting for energy intake. However mean intakes were still 133–156% higher than the recommended target of <5 g salt (<1938 mg sodium) [23] or <4 g salt (<1550 mg sodium) per day for primary and secondary prevention of CVD respectively [24]. This result was surprising given the known association between high sodium intake and CVD and so we would expect that sodium intakes in the CVD group would be either comparable or higher than the group with CVD risk. These results are similar to research conducted by Delaney et al. [25] who found that there was no difference in sodium intake between adults with intermittent claudication (IC) and matched controls. We could speculate that the presence of CVD risk factors or established CVD may lead to individuals paying attention to their sodium intake, however further research is needed to investigate this.

Similarly, there were no differences in the proportion of adults meeting dietary recommendations except for sodium and fibre. A higher proportion of adults in the CVD risk factors and/or established CVD group met sodium recommendations. While the CVD risk factors and/or established CVD group had a lower average intake of sodium, the proportion meeting the recommendations for sodium was low for those at risk or with CVD and the entire sample more generally. It is well established that an increased intake of sodium is associated with raised blood pressure and increases the risk CVD. A meta-analysis conducted by Strazzullo et al. concluded that a higher salt intake was associated with a 23% increased risk of stroke and a 17% increased risk of CVD and that a population-wide reduction in salt intake was warranted [26]. Our results show that adults in Australia are consuming above the recommended intakes of sodium regardless of level of CVD risk and that this has the potential to further increase the prevalence of CVD in future years.

A 2013 meta-analysis of dietary fibre intake and CVD risk found that dietary fibre intake was inversely associated with CVD with a 9% reduction in risk per 7 g fibre/day and a 16% reduction in risk of coronary heart disease (CHD) per 10 g/day higher fibre intake [27]. In our analysis, we found a lower proportion of adults in the CVD risk factors and/or established CVD group met recommendations for fibre intake compared with those with no CVD risk [9.9% (8.4–11.4%) vs 13.4% (11.3–15.5%)]. Similarly to sodium, despite the differences between the two groups, overall proportions meeting recommendations were low providing further evidence that Australian adults in general are not following recommended dietary practices regardless of CVD risk.

Interestingly, adults in both groups were more successful at meeting the AMDR for total fat intake (20–35% of total energy intake) with over 80% of adults having an intake within the AMDR however this did not equate to adults meeting the recommendations for saturated and trans fat combined, indicating that higher proportions of adult fat intakes are coming from saturated sources as opposed to unsaturated sources.

Recently there has been increased interest in the use of LCN3 fish oils both from fish and in supplemental form, in the management of CVD with evidence supporting LCN3 use as a means for lowering plasma triglyceride levels [28]. LCN3 supplements are heavily promoted in the media and hence there is increased exposure for the general public. When we explored LCN3 intake from food and the use of LCN3 supplements in adults with and without CVD risk factors or established disease we found there was no significant difference between the groups in the average intake of LCN3 from foods or the proportion of adults meeting the LCN3 recommendations. There was also no difference in the proportion of adults consuming supplements and that the proportion of use was small at 15.9% (95%CI 13.9–18.1%) in the no CVD risk group and 17.8% (95%CI 16.6–19.1%) in those with risk factors and/or established CVD. These findings suggest that either the messages surrounding the consumption of fish and the use of LCN3 fish oils are not effective in altering practices for those theoretically with the most to gain in terms of health benefits or that there are barriers to individuals consuming more LCN3 [28]. While there appears to be no literature to the authors knowledge regarding the consumption of LCN3 in supplemental form in the CVD population and potential barriers or enablers, there is some literature investigating the intakes of fish both in the CVD population and more broadly. A study by Pieniak et al. [29] investigating fish consumption in households with and without CVD across Europe found that in some countries (Belgium, Denmark, The Netherlands) a higher proportion of participants with CVD consumed fish ≥2/week but overall the proportions consuming this amount were relatively small at 16–46%. The exception to this was in Spain where 71% of those without CVD and 83% of those with CVD consumed fish ≥2/week which is likely reflective of the Mediterranean style dietary pattern in Spain. Another study conducted by the same research group [30] investigated the public perception of the benefits and risks of fish consumption and found that a high proportion (78–80%) of participants believed fish to be healthy and nutritious and that regular consumption of fish reduced the risk of coronary heart disease (73%). However a study conducted in Norwegian women [31] found lack of fresh fish, poor quality and high price to be barriers to increased fish consumption which potentially explains the disparity between the perceptions of health benefits of fish and the proportions of the public meeting recommended fish intakes. In an Australian study of older adults, similar results were observed regarding the understanding of the health benefits of fish with 84% of participants agreeing that fish/seafood assists in the lowering of cholesterol and blood lipids [32]. However the average consumption of fish was again suboptimal indicating that barriers cited by participants (cost, availability of good quality fish) were an important factor to improving fish consumption, Overall these results indicate that the public understand the health benefits of fish and fish oils but require cost-effective methods of obtaining LCN3. Supplemental LCN3 may offer this alternative, however this is yet to be explored.

The results of this secondary analysis of the intakes of adults shows that as a whole, dietary practices of Australian adults are not consistent with evidence-based recommendations for relevant nutrients [3–7, 18, 23, 24] for the prevention of chronic diseases including CVD. Despite a plethora of information available via varying media the intake of adults with CVD risk factors and/or established CVD is not dissimilar to adults without increased CVD risk which implies that attaining optimal dietary intakes is challenging for all Australians. This is cause for concern as chronic disease has a high cost burden to individuals and the community [33]. The prevalence of chronic disease in Australia is currently unprecedented and without cost-effective strategies to halt the upward trajectory there will be significant implications for health budgets that are already under serious pressure.

This secondary analysis also investigated the dietary intakes of nutrients key in the prevention of chronic disease within children residing with an adult who has CVD risk factors or established disease to investigate whether the CVD risk of the adult influences the dietary intake of the child given the plethora of evidence that shows adult care givers have a strong influence over the dietary practices of children [10–13]. We found that there were no differences in the dietary intakes of children who resided with an adult with CVD risk when compared to other children surveyed across all of the nutrients investigated. This also held true when we investigated the proportion of children who met recommended intakes for the key nutrients. The results for children mirrors that for adults as would be expected, given the evidence that parents influence children’s eating habits [10, 11, 13].

These results would suggest the adult CVD risk does not influence the dietary intake of children residing in the same household.

While this analysis has been conducted using data collected with robust methodology it is not without its limitations. Firstly, the adults with CVD risk factors and/or established CVD is a heterogeneous group in terms of the CVD history and potentially includes individuals with risk factors who have made dietary changes based on a diagnosis of increased risk and individuals who have established disease resultant from a suboptimal diet. Based on the data available it was not possible to confidently divide the group into those with risk factors and those with established disease and so having the group as a whole may have affected the overall results for dietary intakes.

A further limitation relates to the assumptions made regarding the relationships between the adults in this analysis and the children. We cannot be completely confident that children in the “other children” group do not reside with an adult who has CVD risk factors and/or established CVD who wasn’t included in the survey. Also we are unable to ascertain the status of the relationship (eg parent, grandparent, family friend) between the adult and child who reside in the same household.

The term ‘children’ in this analysis includes individuals aged 2–17 and it is likely that adults have less influence on individuals at the upper end of this age group which may have implications on the results. In future research, it would be worthwhile to investigate the dietary intakes of children of different ages.

A further limitation to the veracity of this study is the use of two 24-h dietary recalls for estimating usual food intake. Firstly, 24-h dietary recalls rely on participants’ ability to remember what was consumed in the previous 24 h which often results in under-reporting [34]. Secondly, multiple days records are necessary to adequately estimate usual intakes at an individual level with studies suggesting between four [35] and eight [36] days are required when assessing energy and macronutrient intakes. Although the NCI method is considered a sound method of estimating usual intake distribution for foods/nutrients, this method would not produce a true zero intake (i.e. non-consumption) as the logistic regression used in the modelling did not predict a zero value in the probability of consumption and any ‘zero’ amounts were recoded to half of the minimum amount recorded within the dataset [21].

## Conclusions

The presence of CVD or CVD risk factors did not have a significant impact on the dietary intake of selected nutrients in Australian adults except for sodium, where intake was lower in those with the increased CVD risk/established disease but still in excess of recommendations. Similarly, a higher proportion of adults with increased CVD risk/established disease met the recommendations for sodium intake; however the proportion was low overall in both groups. When examining the proportions of adults meeting recommendations, dietary fibre also emerged as a difference between the groups, however this was in reverse to sodium, with a lower proportion of adults with higher CVD risk meeting recommendations. Overall, the dietary practices of Australian adults are not consistent with recommendations for cardiovascular health and there is an increasing need for successful public health strategies to be implemented in an era of an increasing prevalence of chronic disease. Further research could also be undertaken to explore the facilitators to decreasing dietary sodium in this population as it seems that of all the nutrients, this population has some capacity, motivation or supports to make improvements which might be able to be applied to some of the other selected nutrients.

## Abbreviations

ABS: Australian Bureau of Statistics
ADGs: Australian Dietary Guidelines
AHS: Australian Health Survey
ALA: Alpha-linolenic Acid
AMDRs: Acceptable Macronutrient Distribution Ranges
CVD: Cardiovascular Disease
EPA + DHA: Eicosapentaenoic acid + Docosahexaenoic acid
LCN3: Long Chain Omega 3 fats
NCI: National Cancer Institute
NHF: National Heart Foundation of Australia
NHMRC: National Health and Medical Research Council
NNPAS: National Nutrition and Physical Activity Survey
NRVs: Nutrient Reference Values for Australia and New Zealand
SDTs: Suggested Dietary Targets
SEIFA: Socio-Economic Indexes for Area
